# Laparoscopic versus open distal gastrectomy for locally advanced gastric cancer in middle–low-volume centers in Western countries: a propensity score matching analysis

**DOI:** 10.1007/s00423-020-01951-7

**Published:** 2020-08-04

**Authors:** Giovanni Maria Garbarino, Gianluca Costa, Giovanni Guglielmo Laracca, Giorgio Castagnola, Paolo Mercantini, Massimiliano Di Paola, Simone Vita, Luigi Masoni

**Affiliations:** 1 Department of Medical Surgical Science and Translational Medicine, Sant’Andrea Hospital, Sapienza University of Rome, Via di Grottarossa 1035-39, 00189 Rome, Italy; 2 Genaral Surgery Department, San Pietro Fatebenefratelli Hospital, Via Cassia 600, 00189 Rome, Italy

**Keywords:** Gastric Cancer, Laparoscopic surgery, Postoperative recovery, Long-term oncological outcomes, Middle–low-volume centers

## Abstract

**Background:**

Gastrectomy with D2 lymphadenectomy is the standard treatment for patients with resectable gastric cancer. Laparoscopic distal gastrectomy (LDG) is routinely performed for early gastric cancer, and its indications are increasing even for locally advanced gastric cancer. The aim of this study is to compare two middle–low-volume centers in Western countries experience on LDG versus open distal gastrectomy (ODG) for locally advanced gastric cancer in terms of surgical and oncological outcomes.

**Methods:**

We reviewed the data of 123 consecutive patients that underwent LDG and ODG with D2 lymphadenectomy between 2009 and 2014. Among them, 91 were eligible for inclusion (46 LDG and 45 ODG). After propensity score matching analysis, using a 1:1 case-control match, 34 patients were stratified for each group.

**Results:**

The mean operative time was significantly longer in the LDG group (257.2 vs. 197.2, *p* < 0.001). No differences were observed in terms of intraoperative blood loss, average number of lymph nodes removed, and lymph node metastases. The postoperative morbidity was comparable in the two groups. LDG group had a significant faster bowel canalization and soft oral intake (*p* < 0.001). The 5-year overall and disease-free survival were higher for patients treated by laparoscopy, but the post-hoc subgroups analysis revealed that the advantage of LDG was significant just in N0 and stage IB-II patients, whereas N+ and stage III patient’s survival curves were perfectly superimposable.

**Conclusions:**

LDG for locally advanced gastric cancer seems to be feasible and safe with surgical and long-term oncological outcomes comparable with open surgery, even in medium–low-volume centers.

## Introduction

Gastric cancer represents the fifth most common cancer in the world and the third leading cause of cancer death. In Europe, where no screening programs are foreseen, the diagnosis usually occurs at an advanced stage and 5-year survival is reported at around 25% [1, 2].

Total or distal gastrectomy based on the location of the disease associated with D2 lymphadenectomy is the standard treatment for patients with resectable disease (stage IB-III) [3, 4].

For the treatment of locally advanced gastric cancer, a D2 lymphadenectomy is mandatory and it is recommended to be conducted by experienced surgeons in high-volume centers, especially if performed laparoscopically [5–7].

The traditional open surgical approach still represents the most widespread surgical technique.

Since the first laparoscopic distal gastrectomy has been described by Kitano [8], the technique has widely spread, especially in Eastern countries, where several randomized controlled trials (RCTs) have demonstrated better short-term results than open surgery and comparable overall and disease-specific survival rates for the treatment of early gastric cancer, with [9–11].

The Japanese gastric cancer treatment guidelines described, for the first time in 2014 (ver. 4), the laparoscopic distal gastrectomy (LDG) as one treatment option in general practice for stage I gastric cancer (T1N0M0, T1N1M0, or T2aN0M0) [3].

Nowadays, the indications for LDG are constantly increasing, even for locally advanced gastric cancer, as shown by the short-term results of the Eastern countries multicentric RCTs [10, 12, 13].

Recently, the CRITICS trial results showed that high-volume centers (≥ 21 procedures per year) were associated with higher both overall and disease-free survival, emphasizing the value of centralizing gastric cancer surgeries even in the Western world [14]. Conversely, some Korean studies revealed that LDG in low-volume centers is feasible and safe; nonetheless, the early surgical outcomes of LDG performed by the same surgeon in two different volume centers seems to be better when carried out in the high-volume one [15–17].

The aim of this study is to compare LDG and ODG with D2 lymphadenectomy for locally advanced gastric cancer in terms of surgical safety and feasibility, postoperative outcomes, and long-term oncological outcomes in the setting of a middle–low-volume center.

## Methods

### Patient’s selection

**Fig. 1:**
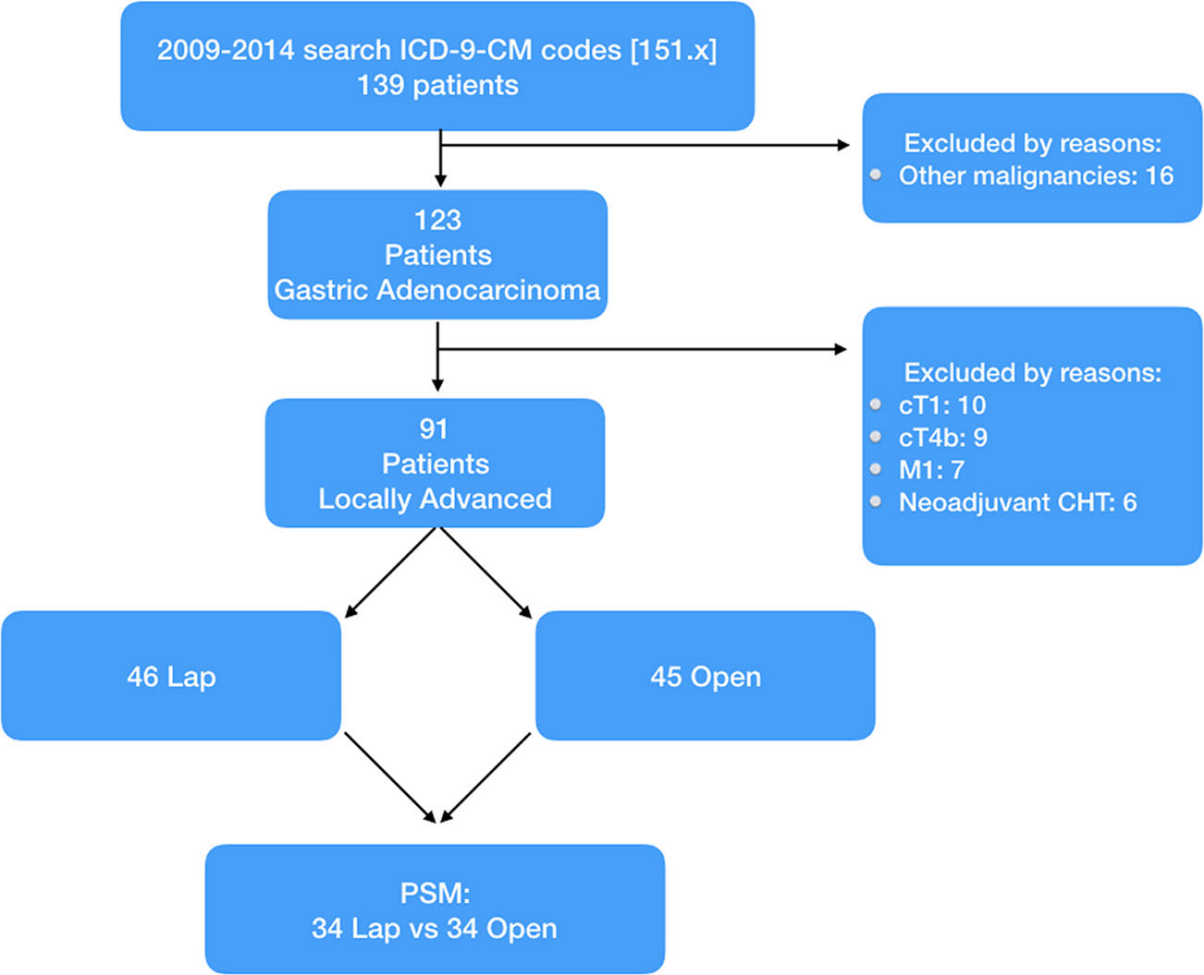
Clinical study design and propensity score method

This was a retrospective case-matched observational study including all consecutive patients undergoing surgery for locally advanced gastric cancer (cT2-T4a, cN0-N3, M0). The exclusion criteria were cT1 and cT4b gastric cancer, metastatic patients, and patients treated by neoadjuvant chemotherapy [Fig. 1]. Distal gastrectomy with D2 lymphadenectomy was performed by two senior staff surgeons (L.M. and S.V.) with large experience in minimally invasive surgery at the Department of General Surgery of San Pietro Fatebenefratelli Hospital and Sant’Andrea Hospital in Rome, between 2009 and 2014.

Indications for surgery were defined on a histologically proven gastric adenocarcinoma of the antro-pyloric region or distal body of the stomach, pre-operative CT staging, anaesthesiologist, and multidisciplinary team evaluation (oncologist, surgeon, radiotherapist, gastroenterologist, pathologist, and radiologist).

Adjuvant chemotherapy (oxaliplatin and capecitabine) was administered according to the “Associazione Italiana di Oncologia Medica” (AIOM) guidelines at time of the surgical treatment (www.aiom.it).

Data was retrospectively reviewed from prospectively maintained database. Data included demographic variables, tumor characteristics and management, operative data, tumor pathology, and short- and long-term outcomes.

Patients were divided into two groups according to the surgical approach: laparoscopic (LDG) and open (ODG) distal gastrectomy.

This study was conducted in accordance with the Declaration of Helsinki and its later amendments. A formal Institutional Review Board approval was not required because of the non-interventional retrospective design; however, a signed consent for the treatment and the analysis of data for scientific purpose was obtained from all patients before any surgical procedures.

### Surgical technique

#### Laparoscopic distal gastrectomy

The patient is placed on the operating table in the French position, with the primary surgeon placed between the legs of the patient, the first assistant on the right side of the patients, and the second assistant on the left side.

Under general anesthesia, 12 mmHg pneumoperitoneum was induced with open laparoscopy approach. Four or five trocars were inserted in the upper abdomen. The first step consisted into the exploration of the abdominal cavity, aimed at excluding the presence of ascites, peritoneal carcinosis, or any metastasis, not identified preoperatively and performing the peritoneal lavage, as previously described [18]. Therefore, the liver, the mobility or the fixity of the gastric lesion, the epiploon, the colon, and the transverse mesocolon are explored to detect any metastases or neoplastic infiltrations. Once the effective resectability of the neoplasm has been established, the operative technique was similar to open surgery: colon-epiploic dissection, access to the epiploon retrocavity, dissection, ligation and section of the right gastroepiploic vessels, section of the hepatoduodenal ligament, followed by ligation, and section of the right gastric vessels. Then the duodenum was divided using a 60-mm endoscopic linear stapler and a D2 lymphadenectomy (1, 3, 4sb, 4d, 5, 6, 7, 8a, 9, 11p, 12a) was carried out en bloc with the specimen with the ligation and section of the left gastric vessels. Gastric resection was performed using a 60-mm endoscopic linear stapler. The resected specimen is placed in a plastic bag and removed preferentially enlarging a 12 mm port. This incision can be used to perform the extracorporeal jejunojejunal anastomosis. Then the abdomen is reinsufflated to perform the laparoscopic Roux en Y gastro-jejunal anastomosis, using a 60-mm endoscopic linear stapler. The enterotomy is closed with a 3/0 absorbable self-locking suture.

The anastomosis can be performed either antecolic or transmesocolic, according to surgeon’s preference.

Abdominal drainages are placed through the trocar orifices: one close to the duodenal stump and the other posterior to the gastrojejunal anastomosis.

#### Open gastrectomy

The patient is placed on the operating table in supine position, closed legs, and arms along the body.

The primary surgeon is positioned to the right side of the patient, the first assistant in front of the surgeon, and the second assistant on the left side of the primary surgeon.

The same steps as for laparoscopic approach were carried out through a midline xifo-umbilical laparotomy. Usually, a transmesocolic gastrojejunal Roux en Y anastomosis was performed with a 60-mm linear stapler and a jejunojejunal anastomosis was performed manually in single layer.

Abdominal drainages are placed close to the duodenal stump and posterior to the gastrojejunal anastomosis.

### Study criteria

Tumor staging was performed according to 7th TNM edition [19]. Pathological data also included the completeness of resection, the lymph node harvest (LNH), and the lymph node ratio (LNR).

Morbidity and mortality were respectively defined as postoperative complications and death within 90 days after surgery. Postoperative morbidity was graded according to the Clavien-Dindo classification [20], in which grade III and IV were defined as “major complications.” Reoperation was defined as every surgical procedure following primary surgery during hospitalization or within 30 days after primary intervention.

### Statistical analysis

The patient baseline characteristics were expressed as the mean (± standard deviation) or median (range) for continuous data and as numbers with percentages for categorical data. Comparison of categorical variables was performed using the *χ*^2^ test or Fisher’s exact test with Yates correction when appropriate. Unpaired Student *t* test was used to compare differences in continuous parametric variables and the Mann–Whitney *U* test for continuous nonparametric variables when appropriate.

In order to compare perioperative and oncological outcomes of laparoscopic and open surgery for gastric cancer, we performed a propensity score matching analysis.

Propensity scores were calculated by bivariate logistic regression, including the following variables that might be considered as potential baseline confounders between the groups: age, Sex, BMI, ASA score, comorbidity, histotype. The exact matching for N stage was also applied. We matched propensity scores 1:1 with the use of the nearest neighbor methods without replacement by using a 0.3 calipers width to achieve the maximum number of cases without statistical differences in confounders variables.

Survival analyses were conducted using the Kaplan–Meier method with log-rank test and Cox regression analysis. Significance was defined as a *p* value of less than 0.05. The statistical analysis was performed using the SPSS version 25.0 (SPSS, Inc., Chicago, IL).

## Results

### Patients’ characteristics

In our study, a total of 123 patients underwent LDG or ODG for gastric adenocarcinoma. Sixty patients were treated with a laparoscopic approach; the other 63 were treated with a laparotomic approach. Of these, 91 patients were eligible for the study because they were affected by locally advanced disease (cT2-4a cN0-3M0): 46 patients underwent LDG and 45 ODG. Patients with cT1, cT4b, metastatic disease, or treated by neoadjuvant chemotherapy were excluded from the study.

Demographic characteristics are shown in Table 1.

**Table 1:** Demographics characteristics before and after propensity score matching analysis

	Before propensity score matching			After propensity score matching		
	ODG	LDG	*p*	ODG	LDG	*p*
	*n* = 45	*n* = 46		*n* = 34	*n* = 34	
AGE (years, mean, ± SD)	72.1 (± 10.1)	72.2 (± 9.9)	0.937	71.1 (± 9.1)	70.9 (± 10.7)	0.951
SEX (*n*, %)			0.608			0.612
M	26 (57.8%)	29 (63.0%)		21 (61.8%)	23 (67.6%)	
F	19 (42.2%)	17 (37.0%)		13 (38.2%)	11 (32.4%)	
BMI (mean, ±SD)	23.3 (± 3.7)	24.2 (±3.8)	0.266	24.2 (± 3.2)	24.2 (±4.1)	0.932
ASA SCORE (*n*, %)			0.063			0.746
1	5 (11.1%)	1 (2.2%)		1 (2.9%)	1 (2.9%)	
2	16 (35.6%)	14 (30.4%)		14 (41.2%)	10 (29.4%)	
3	23 (51.1%)	24 (52.2%)		18 (52.9%)	21 (61.8%)	
4	1 (2.2%)	7 (15.2%)		1 (2.9%)	2 (5.9%)	
Comorbidity (*n* pts, %)	38 (84.4%)	37 (80.4%)	0.615	30 (88.2%)	30 (88.2%)	1.000
Associated disease (*n*, %)			0.734			0.994
Cardiovascular	26 (57.7%)	27 (58.7%)		20 (58.8%)	18 (52.9%)	
Respiratory	11 (24.4%)	9 (20.0%)		8 (23.5%)	7 (20.5%)	
Diabetes	8 (17.8%)	6 (13.0%)		6 (17.6%)	5 (14.7%)	
Chronic renal failure	3 (6.7%)	2 (4.3%)		1 (2.9%)	1 (2.9%)	
Other	4 (8.9%)	1 (2.2%)		2 (5.9%)	1 (2.9%)	
Histotype			0.525			0.353
Intestinal	31 (72.1%)	33 (71.7%)		25 (73.5%)	23 (67.6%)	
Diffuse	10 (22.2%)	12 (26.1%)		6 (17.6%)	10 (29.4%)	
Mixte	1 (2.2%)	0 (0.0%)		0 (0.0%)	0 (0.0%)	
Other	3 (6.7%)	1 (2.2%)		3 (8.8%)	1 (2.8%)	
Tumor size (cm, mean, ± SD)	4.1 (± 1.8)	4.4 (± 2.1)	0.693	4.1 (± 1.9)	4.0 (± 2.2)	0.899

After propensity score matching, two groups of 34 patients, perfectly superimposable as regards the N stage and homogeneous for sex, age, BMI, ASA score, comorbidities, histotype, and tumor size, were extracted.

### Intraoperative characteristics and postoperative outcomes

The intraoperative characteristics and postoperative outcomes are shown in Table 2. Conversion during LDG occurred in 5 (14.7%) patients: because of tumor infiltration of the pancreatic capsule or anterior layer of transverse mesocolon (3 patients) and to obtain a correct surgical exposition (2 patients).

**Table 2:** Intraoperative and postoperative outcomes before and after propensity score matching analysis

	Before propensity score matching			After propensity score matching		
	ODG	LDG	*p*	ODG	LDG	*p*
	*n* = 45	*n* = 46		*n* = 34	*n* = 34	
Operative time (min, mean ± SD)	199.8 ± 61.6	247.3 ± 50.7	< 0.001	197.2 ± 66.4	257.2 ± 46.3	< 0.001
Conversion (*n*, %)		5 (10.8%)			5 (14,7)	
Blood loss	200.1 ± 259.0	153.8 ± 256.2	0.184	180.3 ± 165.3	140.8 ± 170.9	0.217
Time to bowel canalization (days, mean ± SD)	5.8 ± 1.8	3.9 ± 1.3	< 0.001	5.6 ± 1.5	4.1 ± 1.5	< 0.001
Time to soft oral intake (days, mean ± SD)	7.5 ± 4.4	4.7 ± 1.4	< 0.001	7.5 ± 4.8	4.8 ± 1.5	< 0.001
Length of stay (days, mean ± SD)	15.2 ± 12.6	11.6 ± 8.9	0.073	15.8 ± 13.7	11.8 ± 8.3	0.120
Peri-operative complication (*n* pts, %)	16 (35.6%)	8 (17.4%)	0.084	10 (29.4%)	5 (14.7%)	0.144
Clavien-Dindo classification (*n* pts, %)						
I–II	9 (20.0%)	5 (10.9%)	0.227	5 (14.7%)	5 (14.7%)	1.000
III–IV	7 (15.6%)	3 (6.5%)	0.168	6 (17.6%)	2 (5.9%)	0.132
Postoperative complication (*n*, %)						
Anastomotic leakage	1 (2.2%)	2 (4.3%)	0.984	1 (2.9%)	1 (2.9%)	0.473
Duodenal leakage	4 (8.9%)	1 (2.2%)	0.344	3 (8.8%)	2 (5.9%)	0.642
Bleeding	2 (4.4%)	1 (2.2%)	0.984	1 (2.9%)	1 (2.9%)	0.473
Canalization delay	4 (8.9%)	0 (0.0%)	0.056	2 (5.9%)	2 (5.9%)	0.606
Cardiovascular	1 (2.2%)	0 (0.0%)	0.494	1 (2.9%)	0 (0.0%)	0.500
Pulmonary	3 (6.7%)	1 (2.2%)	0.593	2 (5.9%)	0 (0.0%)	0.119
Urinary	0 (0.0%)	1 (2.2%)	0.505	0 (0.0%)	2 (5.9%)	0.119
Other	2 (4.4%)	2 (4.4%)	0.624	2 (5.9%)	2 (5.9%)	0.606
Re-operation (*n*, %)	7 (15.6%)	2 (4.3%)	0.073	6 (17.6%)	2 (5.9%)	0.132
Mortality (*n*, %)	0 (0.0%)	1 (2.2%)	0.320	0 (0.0%)	1 (2.9%)	0.314

The only death occurred as a result of postoperative hemoperitoneum on postoperative day (POD) 1 after LDG for a stage IIIb gastric cancer. The patient underwent reoperation by laparotomy but then developed heart failure.

Briefly, statistical differences were found in the time of surgery (257.2 ± 46.3 vs. 197.2 ± 66.4 min, *p* ≤ 0.001), time to first flatus (4.1 ± 1.5 vs. 5.6 ± 1.5 days, *p* ≤ 0.001), and time to soft oral intake (4.8 ± 1.5 vs. 7.5 ± 4.8 days, *p* ≤ 0.001).

Even if an advantageous trend has been observed for the laparoscopic approach, no statistical differences were recorded about blood loss (140.8 ± 170.9 vs. 180.3 ± 165.3 mL, *p* = 0.217) and time of hospital stay (11.8 ± 8.3 vs. 15.8 ± 13.7 days, *p* = 0.120).

Focusing on the postoperative morbidity, there were no statistical differences between the two groups. Furthermore, the patients treated by open approach developed a higher rate of major complications with the need of reoperation (5.9% vs. 17.6%, *p* = 0.132).

### Pathological and long-term postoperative outcomes

The pathological outcomes are shown in the Table 3. All cytological examinations performed on the peritoneal lavage resulted as negatives. TNM stage (*p* = 0.392), the number or retrieved lymph nodes (24.5 vs. 24.0, *p* = 0.849), and R0 resection (100% vs. 94.4%, *p* = 0.473) were comparable between the two groups. As a result of the propensity score matching analysis, the N stage was perfectly superimposable between the two groups (*p* = 1.000). Nonetheless, the number of metastatic lymph nodes (5.6 ± 5.9 vs. 7.2 ± 7.0, *p* = 0.452) and lymph node ratio (0.2 ± 0.2 vs. 0.3 ± 0.3, *p* = 0.216) were higher in the ODG group, even if not statistically significative.

**Table 3:** Oncological outcomes before and after propensity score matching analysis

	Before propensity score matching			After propensity score matching		
	ODG	LDG	*p*	ODG	LDG	*p*
	*n* = 45	*n* = 46		*n* = 34	*n* = 34	
T-stage (*n*, %)			0.743			0.589
pT1	0 (0.0%)	0 (0.0%)		0 (0.0%)	0 (0.0%)	
pT2	13 (28.9%)	12 (26.1%)		10 (29.4%)	9 (26.5%)	
pT3	17 (37.8%)	21 (45.7%)		13 (38.2%)	17 (50.0%)	
pT4	15 (33.3%)	13 (28.3%)		11 (32.4%)	8 (23.5%)	
N-stage (*n*, %)			0.578			1.000
pN0	7 (15.6%)	12 (26.1%)		7 (20.6%)	7 (20.6%)	
pN1	14 (31.1%)	16 (34.8%)		8 (23.5%)	8 (23.5%)	
pN2	9 (20.0%)	8 (17.4%)		9 (26.5%)	9 (26.5%)	
pN3	15 (33.3%)	10 (21.7%)		10 (29.4%)	10 (29.4%)	
M-stage			1.000			1.000
pM0	45 (100%)	46 (100%)		34 (100%)	34 (100%)	
pM1	0 (0.0%)	0 (0.0%)		0 (0.0%)	0 (0.0%)	
Retrieved nodes (*n*)			0.281			0.849
(mean ± SD)	27.6 ± 16.3	24.6 ± 10.3		26.1 ± 12.3	26.0 ± 10.6	
(median, range)	24.0 (2–94)	24.5 (5–54)		24.0 (2–63)	24.5 (9–54)	
Positive nodes (mean ± SD)	8.8 ± 12.8	5.3 ± 6.2	0.102	7.2 ± 7.0	5.6 ± 5.9	0.452
Node ratio (mean ± SD)	0.3 ± 0.3	0.2 ± 0.2	0.063	0.3 ± 0.3	0.2 ± 0.2	0.216
R0 resection (*n,* %)	43 (95.6%)	45 (97.8%)	0.985	33 (94.4%)	34 (100%)	0.473
TNM stage (*n*, %)						
IA	0 (0.0%)	0 (0.0%)	0.241	0 (0.0%)	0 (0.0%)	0.392
IB	2 (4.4%)	4 (8.7%)		2 (5.9%)	2 (5.9%)	
IIA	6 (13.3%)	9 (19.6%)		5 (14.7%)	6 (17.6%)	
IIB	8 (17.8%)	15 (32.6%)		5 (14.7%)	12 (35.3%)	
IIIA	10 (22.2%)	4 (8.7%)		8 (23.5%)	4 (11.8%)	
IIIB	10 (22.2%)	7 (15.2%)		7 (20.6%)	6 (17.6%)	
IIIC	9 (20.0%)	7 (15.2%)		7 (20.6%)	4 (11.8%)	
IV	0 (0.0%)	0 (0.0%)		0 (0.0%)	0 (0.0%)	

**Fig. 2:**
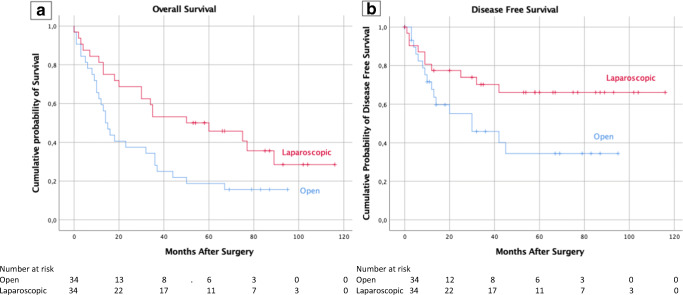
**a** Patient’s overall survival curves according to surgical approach. **b** Patient’s disease-free survival curves according to surgical approach

Focusing on the long-term oncological outcomes, the median follow-up period was 31 months (range 0–116) months; the 5-year overall survival (OS) rate was 26.6% with a mean OS time of 46.2 ± 5.5 months (95% CI 35.4–57.0 months). The 5-year OS was 18.8% versus 45.8%, *p* = 0.018 (Fig. 2).

The 5-year disease-free survival (DFS) rate was 59.4% with a mean DFS time of 68.6 ± 6.9 months (95% CI 55.1–82.1 months). The 5-year DFS was 34.4% versus 66.1%, *p* = 0.044 (Fig. 2).

#### Post-hoc subgroups analysis

We identified two subgroups for long-term outcomes analysis: the nodal status group and the TNM stage group. The first group accounted for 14 (20.6%) patients with N0 disease and 54 (79.4%) patients with N+ disease. Each group had the same number of patients regarding the surgical approach (N0: 7 LDG vs 7 ODG; N+: 27 LDG vs. 27 ODG; *p* = 1.000), resulting from the exact matching on N stage for the propensity score matching analysis. The TNM stage group had 32 (47.1%) patients whit stage IB-II gastric cancer (20 LDG vs 12 ODG) and 36 (52.9%) with stage III disease (14 LDG vs 22 ODG); nonetheless, the difference between the two surgical approaches was not significant, *p* = 0.052.

**Fig. 3:**
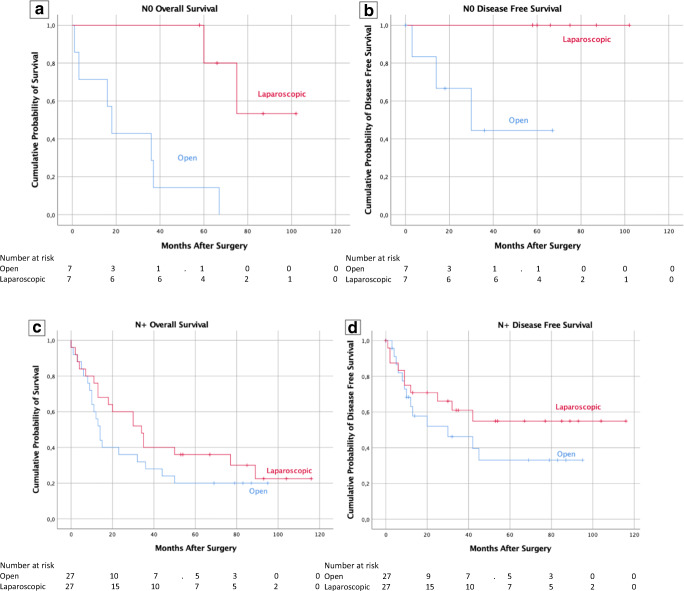
**a**, **b** Patient’s overall and disease-free survival in patients with N0 gastric cancer according to surgical approach. **c**, **d** Patient’s overall and disease-free survival in patients with N+ gastric cancer according to surgical approach

Concerning the nodal status subgroup, we observed statistically significant advantage of laparoscopic approach regarding both 5-year OS (71.4% vs. 0.0%, *p* = 0.001) and DFS (100% vs. 57.1%, *p* = 0.030) only for N0 patients, whereas survival curves for N+ patients were comparable between the LDG and ODG: 5-year OS (28.0% vs. 20.0%, *p* = 0.242) and DFS (60.0% vs. 48.0%, *p* = 0.247) (Fig. 3).

**Fig. 4:**
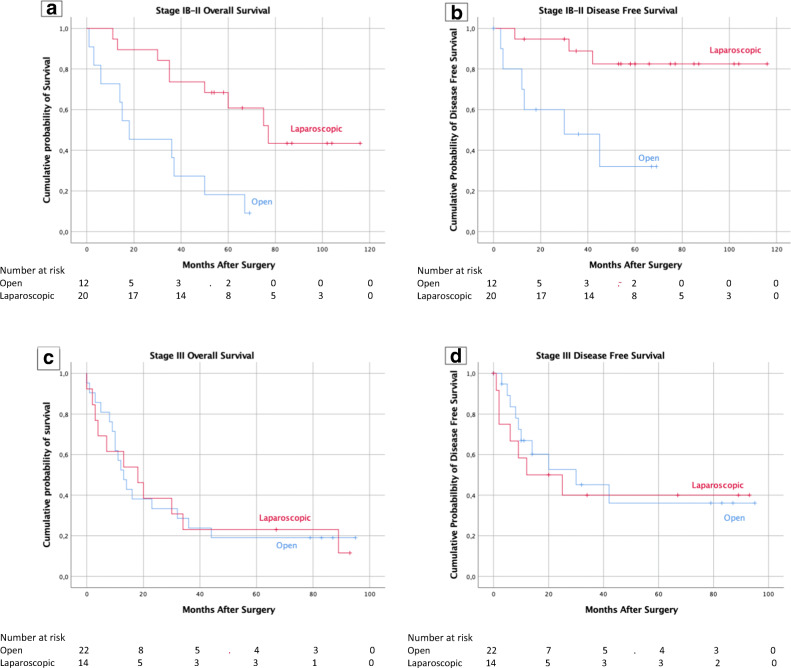
**a**, **b** Patient’s overall and disease-free survival in patients with stage IB-II gastric cancer according to surgical approach. **c**, **d** Patient’s overall and disease-free survival in patients with stage III gastric cancer according to surgical approach

The same result appears in the TNM stage subgroup: stage IB-II patients had a better survival when treated by laparoscopy (5-year OS: 52.6% vs. 9.1%, *p* = 0.002; 5-year DFS 84.2% vs. 45.5%, *p* = 0.005); although, the Kaplan -Meier’s curves of the stage III patients were perfectly superimposable between the two groups (5-year OS: 15.4% vs. 19.0%, *p* = 0.985; 5-year DFS: 46.2% vs. 52.4%, *p* = 0.724) (Fig. 4).

## Discussion

Since the publication of the results of the randomized controlled phase III “AIO-FLOT-4” trial, the gold standard of treatment in Europe for locally advanced gastric cancer (stage IB-III) is radical gastrectomy with D2 lymphadenectomy and perioperative chemotherapy [4].

The minimally invasive approach, despite a longer operative time compared to open surgery, offers advantages in terms of blood loss, postoperative morbidity, faster recovery of intestinal function and oral intake, earlier mobilization, shorter hospitalization, and lower inflammatory response to intervention; all those elements probably contribute to a significant reduction in morbidity, especially related to medical causes [11, 13, 21–24].

Consequently, the Japanese gastric cancer treatment guidelines described, for the first time in 2014 (ver. 4), the LDG as one treatment option in general practice for stage I gastric cancer (T1N0M0, T1N1M0, or T2aN0M0) [3].

Recently, the results of the Korean KLASS-02, Japanese JLSSG0901, and Chinese CLASS-01 RCTs did not show any significant difference in terms of short-term efficacy between open and laparoscopic surgery in the treatment of locally advanced gastric cancer [10, 12, 13].

This study confirmed a significant faster time of bowel canalization and oral intake for patients treated by laparoscopy and showed a favorable trend concerning the blood loss and the hospital stay for LDG group, even if statistical significance was not achieved.

Differences in postoperative morbidity and 30-day postoperative mortality were not seen between the two groups. The only death that occurred in the current series was due to a postoperative hemoperitoneum. The patient underwent reoperation by laparotomy but then developed heart failure.

Focusing on the postoperative morbidity, the patients treated by open approach developed a higher rate of major complications with the need of reoperation.

Although the benefits of LDG in gastric cancer surgery are widely accepted, technical difficulty still represents the main concern of the surgeons [25]. LDG are currently performed by experienced surgeons in high-volume centers. Recently, the CRITICS trial results showed that high-volume centers (≥ 21 procedures per year) were associated with higher both overall and disease-free survival, emphasizing the value of centralizing gastric cancer surgeries in the Western world [14]. On the other hand, some Korean studies revealed that LDG in low-volume centers is feasible and safe [15, 16]. Interestingly, Kim et al. showed that the early surgical outcomes of LDG performed by the same surgeon in two different Korean hospital setting seems to be better when carried out in the high-volume center (> 1000 laparoscopic gastrectomies per year) [17].

In Italy, the minimum number of cases to define a high-volume center for gastric surgery is about 25–40 cases per year, according to Italian Ministry of Health (www.oncoguida.it) and “Programma Nazionale Esiti (PNE)” [26], but no cut-off number of procedures per single surgeon has been established yet.

In our experience, surgery was performed by two oncological surgeons with large experience in open gastric surgery and laparoscopic surgery for colorectal cancer [24, 27–31]. According to a literature review, the learning curve for LDG is not well defined, ranging from 20 up to 100 cases [32, 33].

Focusing on the extension of the lymph node dissection, nowadays the D2 lymphadenectomy with preservation of the pancreas and spleen is considered the standard of care by the guidelines of various global scientific societies [34, 35] and it is recommended to be conducted by an experienced surgeon, especially if performed laparoscopically [6, 7].

In general, laparoscopic D2 lymphadenectomy in locally advanced gastric cancer is believed to be more difficult to perform, as due to several limitations such as bulky tumor with a reduced exposure of the surgical field and determination of correct dissection plane, lesser degree of freedom, unsecure bleeding control, and easier tear of soft tissue [36].

In the current series, all patients underwent a D2 lymphadenectomy with preservation of the pancreas and spleen. The average number of lymph nodes removed was above the minimum number recommended by Japanese and Western guidelines [3, 4], showing how an expert laparoscopic surgeon can perform a D2 lymphadenectomy without any differences in extension and accuracy with open surgery [37].

The results of oncological outcomes showed a 5-year survival rate comparable with the European estimates [1, 2]. When comparing the different surgical approaches, LDG showed an improved OS and disease-free survival DFS rate comparing to ODG. Possible biases such as age, comorbidities, histotype, and nodal stage were included as covariates in the propensity score matching analysis. The number of retrieved lymph nodes were comparable between the two groups, but the number of metastatic lymph nodes and the lymph node ratio were higher in the ODG group, even if not statistically significant.

In order to try to explain this surprising result, we identified two different subgroups analysis: nodal status (N0, N+) and TNM stage.

Nodal stage has been established as an independent predictor of survival in several studies [38–40]; TNM stage by definition is associated with different survival rates. The classification of locally advanced gastric cancer includes a heterogeneous group of patients from stage IB to stage IIIC. Consequently, it is often difficult to compare survival curves of such different patients.

The two subgroups of the current survival analysis were N0 vs. N+ and “early advanced” (stage IB-II) vs. “true advanced” (stage III). Basically, the cut-off stage was identified between stage IIB and IIIA.

The advantage of laparoscopic approach regarding the 5-year OS and DFS was only confirmed in the N0 and “early advanced” subgroups; conversely, N+ and stage III patients had superimposable survival curves.

This study has some limitations like the retrospective fashion of the study and the small sample size. However, despite these limitations, there are several important findings that are generated from this study and validated by the propensity score match analysis. This study confirms that laparoscopic surgery for locally advanced gastric adenocarcinoma is safe and effective, showing perioperative results similar to those obtained with the standard open technique.

To the best of our knowledge, this is the first report showing that 5-year OS and DFS of patients with N0 and “early advanced” gastric cancer can be positively influenced by the minimally invasive approach. Furthermore, the long-term oncological outcomes of patients with N+ and “true advanced” disease were comparable as regarding LDG or ODG.

In conclusion, considering the low incidence of gastric cancer in Western countries, prospective studies are necessary to assess the safety of laparoscopic technique even in medium–low-volume centers.
